# Emerging Viruses in the Felidae: Shifting Paradigms

**DOI:** 10.3390/v4020236

**Published:** 2012-02-07

**Authors:** Stephen J. O’Brien, Jennifer L. Troyer, Meredith A. Brown, Warren E. Johnson, Agostinho Antunes, Melody E. Roelke, Jill Pecon-Slattery

**Affiliations:** 1 Laboratory of Genomic Diversity, National Cancer Institute-Frederick, Frederick, MD 21702, USA; Email: warjohns@mail.nih.gov (W.E.J.); slatterj@mail.nih.gov (J.P.-S.); 2 SAIC-Frederick, Inc., National Cancer Institute-Frederick, Frederick, MD 21702, USA; Email: troyerj@mail.nih.gov (J.L.T.); Melody.Roelke-Parker@nih.gov (M.E.R.); 3 Banfield Pet Hospital, 800 NE Tillamook Street, Portland, OR 97213, USA; Email: Meredith.Brown@banfield.net; 4 CIMAR, University of Porto, Rua dos Bragas, 177, Porto 4050-123, Portugal; Email: aantunes@ciimar.up.pt

**Keywords:** FIV, FCoV, FeLV, Felidae

## Abstract

The domestic cat is afflicted with multiple viruses that serve as powerful models for human disease including cancers, SARS and HIV/AIDS. Cat viruses that cause these diseases have been studied for decades revealing detailed insight concerning transmission, virulence, origins and pathogenesis. Here we review recent genetic advances that have questioned traditional wisdom regarding the origins of virulent Feline infectious peritonitis (FIP) diseases, the pathogenic potential of Feline Immunodeficiency Virus (FIV) in wild non-domestic Felidae species, and the restriction of Feline Leukemia Virus (FeLV) mediated immune impairment to domestic cats rather than other Felidae species. The most recent interpretations indicate important new evolutionary conclusions implicating these deadly infectious agents in domestic and non-domestic felids.

## 1. Introduction

The aloof and elusive nature of domestic cats, *Felis catus*, the world’s most popular pet, is endearing to some and exasperating to others. Including feral cats, there are 600 million to one billion domestic cats worldwide, an astounding number for an animal that contributes little to nothing in the way of work, milk or meat to the human endeavor. The domestic cat is the consequence of a remarkable domestication experiment that commenced in near east Asia some 10,000 years ago, when the shy and reclusive desert wildcat (*Felis sylvestris*) gradually morphed into the venerated, audacious, and familiar human pet. Domestication processes began when Neolithic hunter-gatherers settled in agricultural villages in a rich area of the Middle East we call the Fertile Crescent. These first farmers cultivated wild precursors of corn, wheat and barley, while others herded and penned cattle, sheep, goats and even pigs. The domestication strategy fed and clothed many more people than hunters could support. Contemporaneously, the wildcats fed on scraps, befriended the settlers and began a legacy of human companionship that is unprecedented in human civilization [1].

At first glance, the major benefit of cat domestication appears to be human companionship, but this role is rapidly expanding to encompass issues in human health. Viral infectious diseases in cats have patterns of evolution, virulence and pathogenicity that offer strong parallels to related viruses in humans (Table 1). Feline coronavirus (FCoV), common in domestic cats, is a close relative of the human SARS coronavirus that afflicted the world in 2003, when a reported 8096 infections in 23 countries killed 774 people before the outbreak subsided [2]. In the 1960’s, the discovery of feline leukemia virus and its ability to recombine with host cellular oncogenes resulted in a better understanding of numerous feline and human malignancies [3]. Feline Immunodeficiency Virus (FIV), first identified in 1986 as the causative agent of an AIDS-like syndrome in a California cat colony [4], remains a compelling natural model of immunodeficiency pathogenesis, mirroring the HIV-AIDS epidemic that has dominated the past human generation. Add to that feline calicivirus, feline herpes virus, feline foamy virus and panleukopenia parvovirus; cat species provide a panoply of infectious disease models for many devastating human diseases (Table 1).

**Table 1:** Examples of domestic cat viruses with human homologues *.

Feline Virus	Human Homologue
Feline Leukemia Virus (FeLV) [5]	Human T-Cell Leukemia Virus (HTLV) [6]
Feline Immunodeficiency Virus (FIV) [7]	Human Immunodeficiency Virus (HIV-AIDS) [8]
Feline Coronavirus (FCoV) [9]	SARS-Coronavirus (Severe acute respiratory syndrome) [10]
Feline Sarcoma Virus (FSV) [11]	~20 Human Oncogenes [12]
Avian H5N1 Influenza [13]	Avian H5N1 Influenza [14]
Feline Herpes Virus (FHV) [15]	Cytomegalovirus (CMV-retinitis)
Feline Foamy Virus (FFV) [16]	Human Foamy Virus (No pathology) [17]
Feline Calicivirus (FCV) [18]	Human Calicivirus (Diarrhea, vomiting) [19]
Feline Parvovirus (FPV) [20]	Human B19 Parvovirus (Fifth disease) [21]
Feline Morbillivirus (CDV) [22]	Human Morbillivirus (Measles) [23]

* Many references exist for each virus in both cat and human, here we provide single references as examples.

In this review, we will attempt to highlight how recent advances in our understanding of three cat viruses (FCoV, FIV, and FeLV) have revised conventional wisdoms. We illustrate how previous tenets, based on limited available evidence, were revised and amended due to new insights from genetic studies of cat populations.

## 2. Feline Coronavirus (FCoV) Pathogenesis in Domestic Cats

Feline infectious peritonitis (FIP) is a fatal, progressive, and immune-augmented disease of cats caused by infection with feline coronavirus (FCoV). Coronaviruses are enveloped positive-stranded RNA viruses that infect a wide range of vertebrate species [24]. The clinical manifestation of FCoV infection can present either as the pathogenic disease manifestation or feline infectious peritonitis virus (FIPV) or the more common, benign or mild enteric infection (feline enteric coronavirus—FECV—asymptomatic) [25,26]. Although FCoV is common in domestic, feral and non-domestic cat populations world-wide (seroprevalence from 20–100%), less than 10% of FCoV seropositive cats develop FIP [27,28,29]. Cats infected with FCoV that show no evidence of disease are thought to represent chronic carriers of FCoV and may pose an FIP risk to other cats [27,30,31]. 

FIP pathology is characterized typically by severe systemic inflammatory damage of serosal membranes and widespread pyogranulomatous lesions, occurring in lung, liver, lymph tissue, and brain [32]. Evidence suggests that the host immune system is crucial in this pathogenesis; both profound T-cell depletion from the periphery and lymphatic tissues and changes in cytokine expression are observed in end stage FIP [33,34]. 

Viral gene determinants likely play an important role in FCoV pathogenicity and virulence. Coronaviruses are a large family of enveloped, single stranded, positive sense, non-segmented RNA viruses. Characterized by a genome size roughly 30 kb in length, coronaviruses are the largest RNA virus so far described [35]. However, there is no effective treatment, vaccine, nor a diagnostic protocol that can discriminate the avirulent FECV from the pathogenic FIP strains. 

Conventional wisdom accepts the “*in vivo* mutation transition hypothesis” also called the “internal mutation hypothesis” which postulates that viral mutations occur in healthy FCoV infected cats giving rise to virulent virions that spread systemically and lead to FIP pathogenesis [36,37]. Although this hypothesis has been widely cited [9,25,27,30,31,36,37,38,39] the precise nature of the mutation responsible for pathogenesis has never been identified. Various studies have speculated that variants in the spike protein, membrane protein, or NSP 3c [40] allow infection of macrophages, systemic dissemination, and fatal disease manifestation [36,37]. 

An alternative “circulating avirulent and virulent FCoV hypothesis” suggests that distinctive benign and pathogenic strains of FCoV circulate in a population, and those individuals exposed to the virulent strains, with the appropriate predisposition, develop disease sequelae. Virological precedence for this possibility has been reported for dengue fever virus and equine Venezuelan encephalitis virus, both of which demonstrate circulating virulent and avirulent forms [41,42]. 

To test between these alternatives, Brown *et al.* compared the FCoV gene sequence patterns among FIP afflicted cats and FCoV infected asymptomatic cats collected from households in Maryland between 2004 and 2006 [43]. Brown *et al.* [43] reasoned that a phylogenetic analysis of virogene sequences would be informative in discriminating between the two hypotheses as follows: if the “circulating virulent-avirulent FCoV hypothesis” were the case, then a “monophyletic” pattern of FCoV variation would cluster pathogenic cats separately from healthy cats, since a circulating pathogenic virus would have accumulated mutations over time to drift apart from the avirulent FCoV strain. Alternatively if the “*in vivo* mutation hypothesis” were the case, then FCoV from healthy and sick cats would cluster together (paraphyletic inter-mixing) in accordance with geographic locale (*i.e.*, phylogeographic clustering), and not pathogenic virulence.

**Figure 1:**
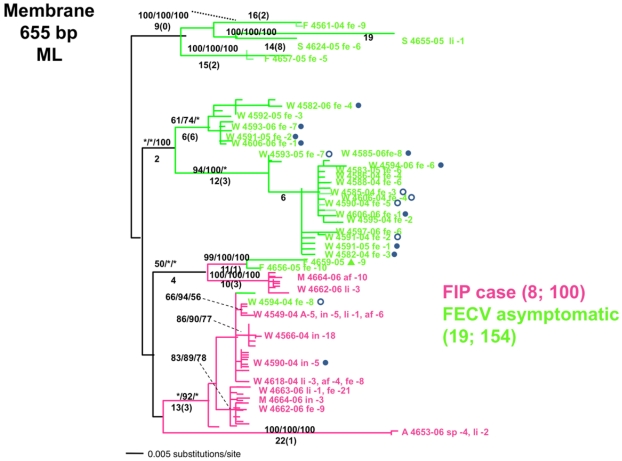
Phylogenetic tree of cloned feline infectious peritonitis (FIP) membrane sequences (655 bp) from 19 healthy (feline enteric coronavirus (FECV); green) and 8 symptomatic (feline infectious peritonitis virus FIPV; red) cats [43]. One cat, Fca-4590, was sampled when healthy and then at death caused by FIP. Shown is the maximum likelihood tree constructed from 655 bp of the membrane gene. The number of FECV and FIP cases followed by the number of cloned sequences is indicated in parenthesis. The labels for each sequence include location W, Weller Farm; F, Frederick Animal Shelter; S, Seymour Farm; M, Mount Airy Shelter; A, Ambrose Farm), 4-digit cat identification number, tissue source (fe, feces; af, ascites fluid; co, colon; li, liver; sp, spleen; in, intestine; je, jejunum; ln, lymph node), 2-digit year (e.g., 04 = 2004), and number of clones for each sequence. Bootstrap values are shown (MP/minimum evolution/ML) above branches. Where ML tree was congruent with MP tree, branch lengths are indicated below branches; the number of homoplasies is in parenthesis after the branch length. Virus sequence obtained from cat no. 4590 in May 2004 and at the time of death due to FIP in December 2004. The transitional individual serial samples are indicated with open circles (first sample) and solid circles (second sample). Scale bar indicates substitutions/site.

Brown *et al.* [43] inspected the phylogenetic clustering of four FCoV genes (*pol replicase*; *spike*, *membrane* and *NSP 7b*; Figure 1) in 56 cats (8 FIP and 46 healthy) from Maryland catteries. The results, illustrated by the phylogenetic analysis in Figure 1, were definitive in clustering the virus from sick FIPV cats together in a monophyletic group, quite distinctive from the FCoV derived from the healthy cats. In one cat (FCA-4590) that progressed from a healthy FECV positive state to FIP disease over the course of two years, the recovered virus from the earlier time point clustered with other FCoV -innocuous strains while the virus isolated when the cat showed disease symptoms clearly grouped with genetically distinct virulent FIP strains, as if the innocuous FCoV were replaced by a second virulent FIPV strain (Figure 1). 

Brown *et al.* [43] interpreted these results as supporting the concept that there were at least two distinctive circulating forms of FCoV in Maryland, one which caused FIP, and a second that did not. Further, they identified certain amino acid signatures of the membrane gene that were diagnostic of FIPV *versus* avirulent circulating FCoV in the feral cat population. Although these results would tend to support the circulating variant explanation, they need to be replicated and extended to other geographic locales to accurately reflect variation possible in the world’s 700 million cats. One recent study documented paraphyly (mixing) of FIP and healthy FCoV infected cat strains in Europe, which would seem to complicate the interpretation [44]. Thus, current evidence would suggest that FIP etiology is more complex than either hypothesis alone would suggest. Perhaps the causative mutation has not been found because multiple mutations may result in increased pathogenicity. It is also possible that some strains are more prone to these mutations than others; a situation that would mimic the circulating avirulent and virulent hypothesis, as well as explain the results seen in Brown *et al.* Future studies exploring both viral and host genetic determinants of disease in FIP [45,46], should reveal opportunities for the management of this disease including the possible development of ante mortem screening tools for genetic disposition for disease as well as the discrimination of virulent *versus* avirulent strains of FCoV.

## 3. Feline Immunodeficiency Virus: FIV Pathogenesis in Felidae Species

Feline Immunodeficiency Virus (FIV) was first discovered 25 years ago [4,47] as a cat lentivirus with structural, genomic, and pathogenic parallels to HIV [48,49,50]. Infected domestic cats develop symptoms of immune depletion including a precipitous drop in CD4 bearing T-lymphocytes, neutropenia, lymphadenopathy and susceptibility to normally harmless bacteria, fungal lesions, wasting, and rare cancers. FIV is endemic in feral cat populations and has diverged into several phylogenetic clade types across the world [51,52,53,54,55]. 

**Figure 2:**
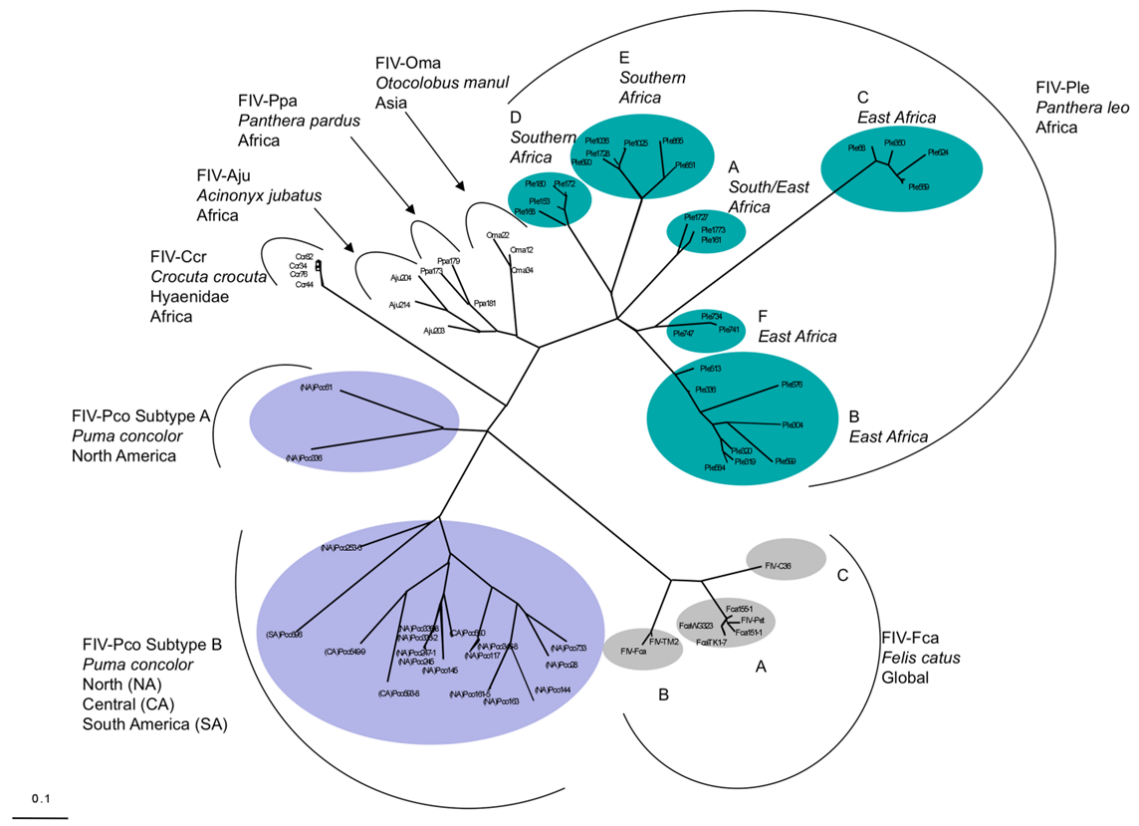
Maximum likelihood phylogenetic tree of 72 non-identical FIV from seven carnivore species based on a region of pol-RT (420 bp) [7,58,65]. Circles indicate subtypes within FIV*_Ple_*, FIV*_Pco_* and FIV*_Fca_* lineages.

FIV has infected many of the 37 described species of the Felidae family [56] (Table 2). It is speculated that most cat species (Table 2) acquired the virus within the last 10–20,000 years, but patterns of evolution within both virus and host genomes, suggest FIV may have existed far longer in some species such as lion [57,58]. Phylogenetic analysis of individual FIV-isolates in a dozen or more species of felids [7,58,59,60,61] demonstrates reciprocal monophyly of FIV among various species (that is, every lion strain has as its closest relative another lion isolate rather than FIV from a different cat species) (Figure 2). These phylogenetic results supported the notion that although FIV occasionally can move from species to species [62,63,64], these events are exceedingly rare, leading to a monophyletic expansion of viral genome sequence diversity within every species, so that most cat species carry their own distinct version of FIV [65,66,67,68].

**Table 2:** Summary of FIV prevalence tested by western blot (AB) and PCR in Felidae.

Species	Common Name	Free Ranging		Captive		Citation
		AB+	PCR+	AB+	PCR+	
*Felis silvestris*	European wild cat	5/125	0/3	4/13		[7,69,70,71,72]
*F. libyca*	African wild cat			1/16	0/1	[7]
*F. bieti*	Chinese desert cat					
*F. margarita*	Desert cat	0/14		6/13	0/7	[7,69]
*F. nigripes*	Black-footed cat			3/11	0/4	[7]
*F. chaus*	Jungle cat			5/17	0/6	[7]
***Otocolobus manul***	**Pallas cat**	**10/27**	**7/26**	**12/19**	**2/2**	[7,43]
*Prionailurus rubiginosis*	Rusty spotted cat			0/1		[7]
***P. bengalensis***	**Asian leopard cat**	**1/12**	**1/1**	0/81		[7,73,74]
*P. viverrinus*	Fishing cat			1/25	0/2	[7]
*P. planiceps*	Flat-headed cat	0/2		1/9		[7]
***Puma concolor***	**Puma**	**150/360**	**61/123**	45/166		[7,61,75,76,77]
*P. yagouaroundi*	Jaguarundi			**9/40**	**1/8**	[7,78]
***Acinonyx jubatus***	**Cheetah**	**22/303**	**7/10**	6/242		[7,75,79,80]
*Lynx pardinus*	Iberian lynx	7/74	0/75			[7,81]
*L. lynx*	Eurasian lynx			0/10		[7,75]
*L. canadensis*	Canada lynx	0/92		1/2	0/1	[7,82]
***L. rufus***	**Bobcat**	**32/115**	**17/32**	1/8	0/1	[7,83]
***Leopardus pardalis***	**Ocelot**	**8/26**	**1/14**	10/88	0/6	[7,75,78]
***L. wiedii***	**Margay**	**1/5**	**1/1**	**4/88**	**1/3**	[7,78]
*L. jacobita*	Andean mountain cat					
*L. colocolo*	Pampas cat			1/12		[7]
*L. geoffroyi*	Geoffroy's cat	1/6	0/1	8/45	0/7	[7]
*L. guigna*	Kodkod			0/2		[7]
*L. tigrinus*	Tigrina			3/40	0/2	[7,78]
*Caracal caracal*	Caracal	0/3		0/22		[7,79]
*C. aurata*	African golden cat			0/2		[7]
*C. serval*	Serval			0/4		[7,75]
*Pardofelis badia*	Bay cat			0/1		[7]
*P. temminckii*	Asian golden cat	0/1		3/29	0/2	[7]
*P. marmorata*	Marbled cat			2/10	0/3	[7]
***Panthera leo***	**Lion**		**212/321**	72/132	1/1	[7,75,80,84,85,86]
*P. onca*	Jaguar		0/2	8/42	0/7	[7,75,78]
***P. pardus***	**Leopard**		**7/10**	1/96	0/1	[7,75,79,80]
*P. tigris*	Tiger	0/1		**25/217**	**1/12**	[7,75]
*P. unca*	Snow leopard			**3/77**	**1/2**	[7,75]
Neofelis nebulosa	Clouded leopard			4/59	0/2	[7]

Bold numbers = congruence between AB and PCR; shaded = free-ranging “+”; bold letters = PCR free-ranging “+” animals.

Originally, the absence of clear clinical pathology among FIV infected felids in zoological collections, and field observations of seemingly healthy (or asymptomatic) FIV in natural populations of felids fostered the view that FIV is pathogenic in domestic cats but not in other free ranging species of Felidae [60,87]. However, that conclusion now seems premature and over-simplified. For example, Roelke *et al.* [88]. mounted a detailed physical examination and associated clinical measures among 64 free ranging lions in Botswana and Tanzania between 1999 and 2006. They examined a suite of biochemical, clinical, and pathogenic manifestations of immune suppression and disease analogous to pathogenesis observed in FIV infected domestic cats, in HIV-infected AIDS patients and in simian immunodeficiency virus (SIV)-infected macaques (see citations [88]). 

Multiple indications and sequelae of AIDS defining conditions were manifest amongst FIV infected lions compared to FIV negative lions; the statistical associations are summarized in Table 3. First, a marked depletion of CD4 bearing T lymphocytes was apparent in FIV infected lions, a prelude to immune collapse in well defined AIDS [88,89]. In addition there were multiple elevations in opportunistic infections (papilloma, gingivitis, dehydration during wet conditions, anemia, hyperalbuminemia, weight loss in the face of abundant prey, abnormal red cell parameters, depressed serum albumin, liver pathogenesis, and elevated gamma globulin). Further, spleen and lymph node biopsies from nine free ranging lions revealed evidence of lymphoid depletion, the hallmark of AIDS disease in human, cats and macaques. These findings strongly suggest FIV is contributing to the loss of immune competence in these lions. A similar pathogenic study of wild SIV-infected chimpanzees also revealed definitive evidence of pathology in that species after a decade of pronouncing chimps as resistant to SIV [90].

As most people infected with HIV do not actually die of HIV infection per se, rather from subsequent opportunistic infections (e.g., pneumocystis, CMV, Kaposi’s sarcoma, candidiasis and other infections) it seemed fair to ask whether FIV in large cats might contribute to secondary infection pathogenesis. An opportunity to inspect this occurred during the mid-1990s in Tanzania when an outbreak of canine distemper virus (CDV; a morbillivirus) eliminated ~1000 lions from the large Serengeti populations in a 10 month interval [91]. Because FIV prevalence in East African and Botswana lions approaches 100% in adults, the potential influence of FIV on CDV pathology was to us an interesting question.

**Figure 3:**
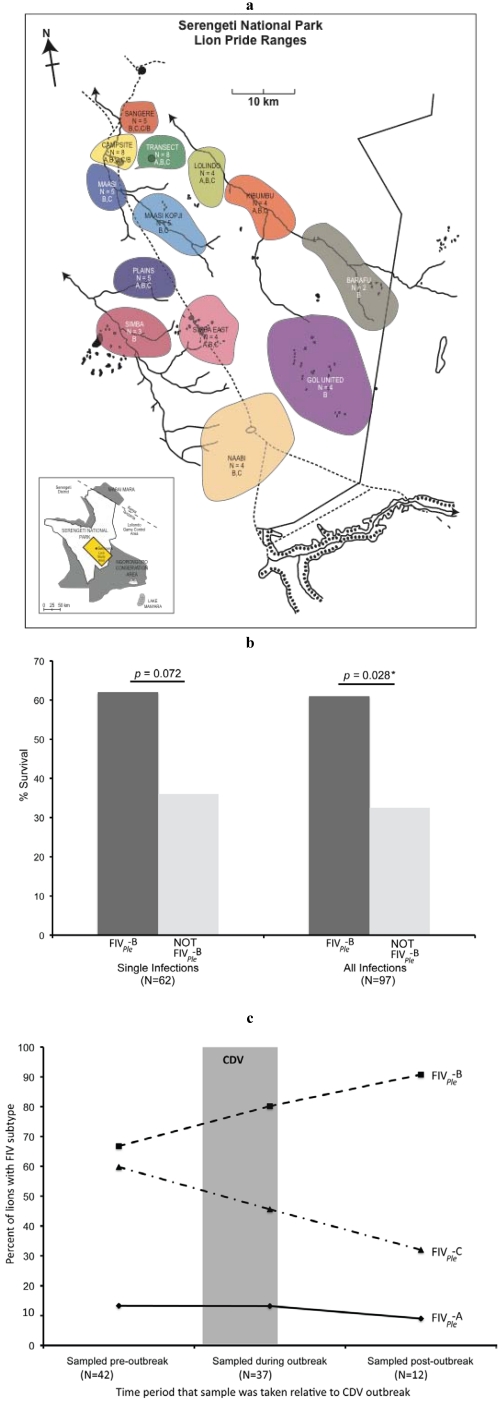
Distribution, incidence and co-infection of canine distemper virus (CDV) and FIV in Serengeti lions. (**a**) Map of approximate pride home ranges during the CDV outbreak in April of 1994. Distribution of FIV*_Ple_* subtypes by pride is shown here [87,93]. (**b**) Comparison of survival between lions with (dark grey) and without (light grey) FIV*_Ple_*-B. Shown here are Chi‑squared p-values. Fisher’s exact two-tailed statistics are significant all subtype configurations (*p* = 0.028) and approaching significant for single subtype infection (*p* = 0.072). (**c**) FIV*_Ple_* subtype distribution over time. Lions that were alive at the beginning of the CDV outbreak (N = 91) were sampled either prior to April 1994, during the month of April 1994, or after April 1994. Most of the 1994 sampling occurred in April after the peak mortality (approximate time shown here as a grey bar). Knowledge of subtype frequencies prior to April 1994 is primarily from samples collected from those animals in previous years. These regressions are significantly different (*p* = 0.001).

Lions harbor six genetically distinct strains, or subtypes, of lion FIV (FIV*_Ple_*) resolved by phylogenetic analyses [57,58] (Figure 2). These strains have distinct phylogeographic distributions, suggesting prolonged host association, perhaps predating the Late-Pleistocene expansions of lions roughly 325,000 years ago [57]. Two lion FIV*_Ple_* strains, FIV*_Ple_* E and FIV*_Ple_* A, circulate in Botswana; while three very divergent strains FIV*_Ple_* A, B, and C occur in the Serengeti [92,93]. Perhaps consequent of the highly social nature of lions, FIV*_Ple_* infected lion populations have high prevalence of seropositive individuals, approaching 100% in adult animals [7,57,92] (Figure 3a). 

Troyer *et al.* [94] recently examined the association of FIV strains with relative survival (from death) in the Serengeti lions during the CDV outbreak. A rather striking difference was seen in that FIV*_Ple_* B infected lions were twice as likely to survive CDV compared to lions infected with alternative strains FIV*_Ple_* A and FIV*_Ple_* C (Figure 3b). The apparent FIV*_Ple_* B associated protective influence was evident whether individuals were infected with a single strain or with multiple strains (Figure 3b). These observations would suggest that infection with FIV*_Ple_* A or C might have increased the risk of mortality upon secondary CDV infection. This inference that certain FIV*_Ple_* strains predispose carriers to CDV pathogenesis has some parallels with FIV strain-specific pathogenicity in domestic cats [95,96,97]. Further, the higher CDV mortality among of FIV*_Ple_* A and C carrying individuals actually altered FIV strain incidence causing a rise in FIV*_Ple_* B and a drop in FIV*_Ple_* C during the course of the CDV outbreak (Figure 3c).

**Table 3:** Medical conditions present in HIV, SIV and FIV infections found in FIV*_Ple_* infected wild lions compared with FIV*_Ple_* negative lions. (Adapted from [48]).

Medical Condition	FIV*_Ple_* Negative		FIV*_Ple_* Positive		Odds Ratio	P Value
	% Affected	# Individuals	% Affected	# Individuals		
**Immunodeficiency**						
CD4 depletion *Absolute number of CD4+ T-cells /mL in peripheral whole blood ±s.e.*	0	5	100	8	NA	0.00015
**Oral manifestations**						
Gingivitis	40	15	88.4	43	11.4	0.00016
Papillomavirus	14.3	14	53.19	47	6.82	0.01009
**Chronic Inflammatory Response**						
Lymphadenopathy	41.67	12	76.6	47	4.58	0.01900
Hyperglobulinemia	0	14	85.71	46	NA	<2 × 10^−9^
Erythrocyte sedimentation rate	13.33	15	64.86	37	12	0.00076
(*> 2 s.d. above mean*)						
Dehydration (> 4%)	26.67	15	63.04	46	4.69	0.01408
**Loss of Condition and Under Nutrition**						
Hair and coat abnormalities	13.3	15	52.27	44	7.12	0.00840
Hypoalbuminemia (marker of cachexia) (*serum albumin > 2 s.d. below mean*)	0	14	46.94	46	NA	0.00129
Anemia (*hemoglobin / PCV >2 s.d. below mean)*	11.11	18	55.77	52	10.09	0.00101
Cachexia/unexplained weight loss	Not documented		Observed in 3 FIV+ populations		NA	NA
**Lymphoid response evidence**						
Histopathologic evidence: Lymphoid activation	Not documented		Yes		NA	NA
Histopathologic evidence: Lymphoid atrophy & depletion	Not documented		Yes		NA	NA

The statistical rigor associated with these conclusions is rather weak since the number of lions was limited (total = 119 lions) and should be interpreted cautiously. Nonetheless, the striking influence of FIV on lion immune function (Table 3), clinical disposition, and a potential ancillary role in CDV mortality (Figure 3b,c) affirms that FIV is likely pathogenic in lions. However, the degree to which viral pathogenicity is influenced by host genomics underlying the immune response, the role of secondary infections, stochastic events due to ecological and environmental factors, has yet to be described. Nonetheless, FIV is a potentially harmful agent in free ranging lions, as for housecats, and deserves further scrutiny in the other free ranging species afflicted with FIV [88,94]. 

## 4. FeLV Outbreak in Free Ranging Florida Pumas

Feline leukemia virus, a retrovirus of domestic cats, displays a prevalence of 1–8% among feral cats worldwide. Transmission is usually by direct contact, and outcome after exposure depends on several host and viral factors. In approximately one third of exposed cats, viremia is persistent and eventually results in clinical syndromes including some combination of immunosuppression, anemia and/or neoplasia [5,98]. Mortality among persistently infected domestic cats is high as 83% die within 3.5 years [99].

Like other Type C retroviruses, FeLV induces immune suppression making the cats susceptible to opportunistic infections and cancers. There are four naturally occurring exogenous FeLV strains FeLV-A, -B, -C, and -T, that are distinguished genetically by sequence differences in the env gene and by receptor interactions required for cell entry [100]. FeLV-A is the predominant subgroup circulating in feral cats and is often only weakly pathogenic [101]. The endogenous feline leukemia provirus sequences are transmitted vertically though the germ line as integrated provirus nested on several cat chromosomes. Among infected cats the pathogenic subgroups, FeLV-B, -C, and -T, are generated *de novo* by mutation or recombination in the env region between exogenous subgroup A virus and endogenous proviral sequences [5,102,103,104].

FeLV infection among non-domestic cats of the Felidae family is rare. Most reported infections involved captive animals that acquired FeLV by physical contact with FeLV-infected domestic cats, and in nearly all cases that were followed, the virus was cleared by the infected individuals [105,106]. Therefore, it was postulated that FeLV pathogenicity did not occur in exotic felids, simply because there were no endogenous FeLV present in species outside the domestic cat lineage. The outcome with a Florida panther FeLV outbreak in 2001–2006 was unexpected and served to change this hypothesis [105,107].

The Florida panther (*Puma concolor coryi*) is an endangered subspecies whose range was contiguous with other puma populations [108]. By the late 20th century, however, depredation, exploitation, human population growth and habitat destruction had reduced the population to an isolated relict population of fewer than 30 individuals [109]. In 1995, a Florida panther restoration management action relocated eight Texas cougars (*Puma concolor stanleyii*) to the Florida habitat in a hopeful rescue of the threatened subspecies. The population rebounded to over 100 individuals, doubling panther numbers, density, survival parameters and fitness [110,111,112].

Florida panthers have undergone continued surveillance from 1978 -2001 and routinely tested for several pathogens, including FeLV [105]. However, for the first time in early 2001, 23 panthers were discovered to carry antibodies for FeLV by ELISA that was confirmed by Western Blot. Clinical symptoms including lymphadenopathy, anemia, septicemia and weight loss rapidly appeared. Five panthers shown to carry FeLV antigens in their sera subsequently died of diseases compatible with FeLV etiology [105,107].

The rapid appearance and spread of FeLV in this Florida panther population was unprecedented among large cats and caused concern in the Felidae conservation community. FeLV was not thought to cause serious disease in species other than in cats closely related to domestic cats (*F. catus*, *F. sylvestris*, *F. margarita*, *F. nigripes and F. bieti*) because only these *Felis* species carry endogenous FeLV sequences in their genome, a prerequisite for *in situ* development of recombinant and virulent FeLV strains [5,102,103,113].

To explore the origins and the unusual virulence of the emerging FeLV strain in pumas, Brown *et al.* [107] obtained infectious FeLV gene sequence (LTR and env genes; 2851 bp) from several FeLV-infected Florida panthers. Alignment and phylogenetic analysis of panther FeLV gene sequences and those from known domestic cat FeLV strains revealed three important aspects: (1) The panther FeLV was clearly aligned with FeLV domestic cat type FeLV-A, the strain that is largely avirulent until after recombination with endogenous sequences; (2) There was no evidence of endogenous FeLV sequences within in the panther FeLV; and (3) The panther FeLV was closely aligned with a highly virulent FeLV from domestic cats, FeLV-945. Although FeLV-9545 is considered an FeLV-A strain, it has a distinctive envelop and LTR sequence that are different from other FeLV-A strains. FeLV-945 is unusual is that its severe pathogenicity in domestic cats does not involve recombination with the endogenous FeLV sequences [114,115]. A vaccination campaign was initiated in 2006 and 52 Florida panthers were captured and vaccinated with no major FeLV incidence reported to date.

An interesting corollary to the Florida panther FeLV outbreak is that FIV*_Pco_* is endemic in this population. Two distinctive strains were present in 2001, one from the original authentic Florida panther and a second accidentally introduced in 1995 from FIV*_Pco_* infected Texas cougars. FIV incidence in the population was low (~15% in 1999–2000; [107]). By contrast, 13 of 17 panthers tested during 2004–2005 in the FeLV-endemic region (76%; Figure 1) were FIV positive [105,107]. This apparent elevation in FIV incidence among FeLV afflicted panthers raises the possibility of a role for FIV-mediated immune depletion in FeLV pathogenesis. In domestic cats, FIV and FeLV co-infections have resulted in conflicting interpretations [116,117,118,119,120,121]. 

The conclusion here is that domestic cat strains of viruses can cross species barriers with potentially devastating consequences to fragile wild populations of large felids. In this case, the requirement for endogenous FeLV recombination was abrogated and perhaps the resultant virulence was accelerated by FIV immune suppression in Florida panthers. As in lions, FIV depletes puma CD4-T lymphocytes [89], so the possibility of FIV accessory role is feasible. Unfortunately, a similar outbreak has recently occurred in wild populations of Iberian lynx [81], confirming that FeLV is capable of causing disease in non-domestic felids, contrary to conventional wisdom.

## 5. Conclusions

Early attempts to characterize the genetics, epidemiology and pathogenicity of feline viruses established the following accepted paradigms in viral disease: that fatal FIP resulted from a simple single mutation from the benign FCoV; that FIV was host-adapted and innocuous in non-domestic felids; and that FeLV-related disease could only occur in species within the domestic cat lineage and always resulted from recombination with endogenous FeLV. However, recent studies augmented by technological advances as well as increased surveillance of free-ranging cat species are revising these perceptions. FCoV strains may have different virulence, pathogenicity, and predisposition to FIP causing mutations; etiologies may be complex and different in different areas, cats may harbor multiple strains throughout their life, and diagnostic genetic profiles may someday be available. FIV in wild African lions, once considered benign, is causally linked with AIDS-related symptoms in some individuals, and some strains may increase susceptibility to co-infection and mortality. Fragile relic populations of Florida panther and Iberian lynx, once thought immune to domestic cat FeLV, are highly susceptible to certain strains that are able to emerge in new host species. Thus, several conventional paradigms have been unseated by recent studies of virus-host interactions in the wild.
